# Do memories of the Ebola virus disease outbreak influence post-Ebola health seeking behaviour in Guéckédou district (epicentre) in Guinea? A cross-sectional study of children with febrile illness

**DOI:** 10.1186/s12889-020-09359-0

**Published:** 2020-08-27

**Authors:** Bienvenu Salim Camara, Junko Okumura, Alexandre Delamou

**Affiliations:** 1 Centre National de Formation et de Recherche en Santé Rurale de Maferinyah, Forécariah, Guinea; 2 School of Tropical Medicine and Global Health, Nagasaki University, Nagasaki, Japan; 3 Institute of Tropical Medicine, Nagasaki University, Nagasaki, Japan; 4 Department of Public Health, Gamal Abdel Nasser University, Conakry, Guinea

**Keywords:** Ebola virus disease (EVD), Febrile illnesses, Health seeking behaviour, Rural, Guinea

## Abstract

**Background:**

The 2013–2015 Ebola Virus Disease (EVD) outbreak in Guinea resulted in community mistrust that influenced health care service utilization. This study aimed to assess whether EVD-related memories affect post-outbreak health-seeking behaviours for children under 5 years of age with febrile illnesses in Guéckédou district, Guinea.

**Methods:**

This cross-sectional study was conducted by surveying caregivers of children under 5 years of age in the sub-district most affected by the EVD outbreak (Guèndembou) and the least affected sub-district (Bolodou) in Guéckédou district. Memories of the outbreak were referred to as EVD-related fears in the post-EVD period, which was based on a series of questions regarding current feelings.

**Results:**

While the majority of caregivers sought care for their children with febrile illness in both districts, a statistically significantly higher proportion of caregivers in Guèndembou sought care, compared to caregivers in Bolodou.. More caregivers in Guèndembou (19.9%; *n* = 39) reported the death of family members or friends due to EVD compared to Bolodou (6.9%; *n* = 14; *P* < 0.001). The mean EVD fear score of caregivers was significantly higher in Guèndembou (3.0; SD: 3.0) than in Bolodou (2.0; SD: 1.1) (*p* < 0.001). Caregivers with a fear score above the median were 1.68 times more likely to seek care than those whose fear score was equal to or below the median; however, this difference was not statistically significant. Caregivers who reported family members’ or friends’ death due to EVD were also more likely to seek care (AOR = 2.12; 95%CI: 0.91–4.91), however, with no statistical significance. Only residing in the EVD-most affected sub-district of Guèndembou (AOR = 1.74; 95%CI: 1·09–2.79) was positively associated with seeking care.

**Conclusions:**

This study reveals that community members in the rural district of Guéckédou still live with fear related to EVD nearly 2 years after the outbreak. It calls for more efforts in the health domain to preserve communities’ key values and address the psychosocial effect of EVD in rural Guinea.

## Background

The 2013–2015 Ebola virus disease (EVD) outbreak was the widest, longest, and deadliest one ever witnessed worldwide [1]. In Guinea, where 3351 EVD cases were reported - out of which 2083 (62·2%) died [2] - the devastating pace of the EVD frightened and confused communities [3, 4].

In fact, the inappropriate approach of the early EVD response, which included poor communication about the disease and lack of involvement of community agencies in response activities at the community level, led to a general misconception of the outbreak and mistrust in the health system [5]. Indeed, the EVD response required measures that were perceived by the community to be provocative and dismissive of traditional values. For instance, despite being part of local communities’ key traditional values, handshaking was discouraged, and community burial or mourning was prohibited [3, 4, 6–8]. Community misconception of the EVD outbreak seemed to cause non-adherence to EVD prevention and control measures. This led to the spread of the outbreak and ravaged families [3, 4, 8].

Further, people refrained from attending health facilities, fearing that they would be considered EVD cases or be contaminated by the virus [5, 9, 10]. The decline in health service utilization may have been more pronounced for febrile illnesses because fever is the main symptom of EVD. This could be particularly detrimental for malaria patients, especially children who are the most predominantly affected group in Guinea [11, 12]. Plucinski et al. reported a 15% decline in public health facility attendance for febrile illnesses and 74,000 fewer malaria cases were treated in Guinea in 2014 compared to 2013 [11].

New approaches, including community involvement in EVD response activities, were later implemented, and they contributed to overcoming the outbreak [8, 10, 13]. For instance, a study in 2015 reported that 62% of community respondents in Guinea interrupted their practice of traditional initiation ceremonies following implementation of a community-based awareness-raising campaign during the EVD outbreak [14].

Although the EVD outbreak is over in Guinea, communities might still suffer fears related to it. Their tragic human experience of the outbreak [5, 8], and the negative effect of the outbreak crisis on their socioeconomic level,[14] might influence their post-outbreak feelings and behaviours, including their health-seeking behaviours (especially for febrile illnesses). Children’s health or chance of survival during early childhood highly depends on appropriate health care service utilisation. Therefore, this age group might be particularly vulnerable to a change in health-seeking behaviour [12].

Little is known about the effects of disease outbreaks on health-seeking behaviour. However, in Mexico, Aguero and Beleche concluded that the 2009 H1N1 pandemic motivated people into changing their behaviour (washing their hands) and this behaviour change led to a decline in diarrhoea cases for children during and 3 years after the pandemic [15]. In Guinea, there is very little information available regarding the influence of EVD on health-seeking behaviour post-outbreak. However, in the post-EVD period, a recovery to pre-EVD levels has been reported in reproductive health services and childhood vaccination in the Forest Guinea region [9, 16, 17]. In Guéckédou district, located in Forest Guinea region, all-cause health service visits for children under 5 years of age were recently reported to have recovered to pre-EVD levels, but the number of malaria cases seen at health centres was still lower than before the EVD outbreak [18]. These studies were based on data collected by health facilities and did not investigate the attitudes and experiences of community members themselves. Information on post-EVD health-seeking behaviour and the reasons why some caregivers do not seek health care for their children is essential to guiding post-EVD interventions for community health in terms of prevention, access to care, and psychological well-being. Therefore, this study sought to assess whether EVD memories among community members influence health-seeking behaviour for febrile illnesses in children under 5 years of age in the post-outbreak period (2016–2017) in rural Guinea.

## Methods

### Study design

This was a survey administered by an interviewer, with closed-ended questions. The survey was part of larger mixed-method study, with the qualitative component expected to be presented elsewhere.

### Study setting

Guinea is located in West Africa and had a population of 10·5 million in 2014. Most of Guinea’s residents are illiterate (67%), live in a rural setting (71%) and subsist below the poverty line (55%) [12, 19]. The country has 33 districts of which 25 were affected by the EVD outbreak. The national health system is tiered in primary, secondary, and tertiary levels [20]. At the primary level, community healthcare workers (CHWs) provide healthcare and prevention services within communities [21].

**Fig. 1:**
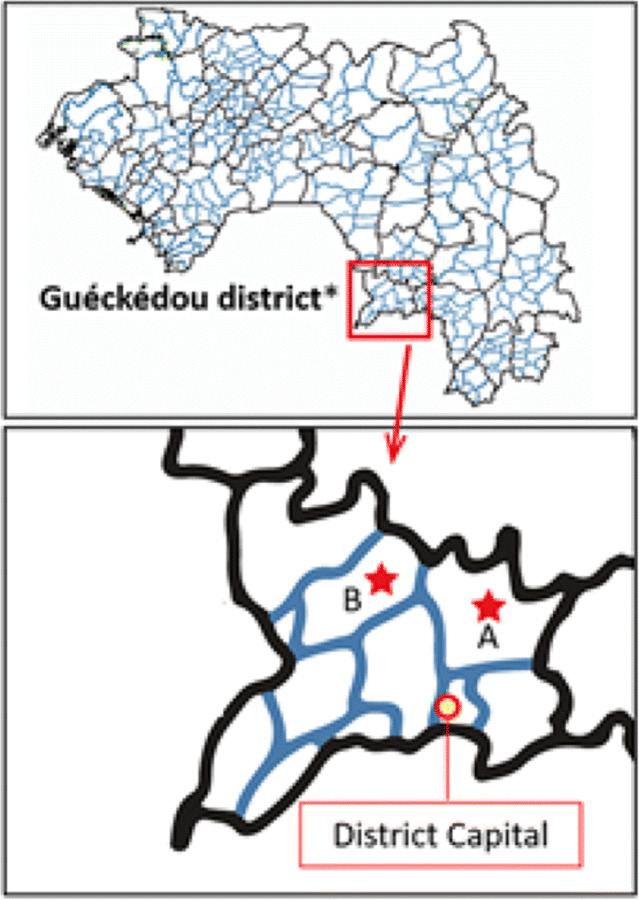
Map of the study setting. Source: Study authors.  Study sites A: Guèndembou (60 EVD cases) B: Bolodou (1 EVD case). * Guéckédou district is an EVD epicentre and prevalence of malaria among children under five years of age in this district was 61% ^12^

The study was conducted in the district of Guéckédou, south-eastern Guinea (Fig. 1). Guéckédou was the epicentre of the EVD outbreak [22] and recorded the highest EVD mortality rate countrywide, with 204 deaths out of 270 confirmed cases (76%) [2]. It was also among the localities that experienced more community resistance to EVD response activities [4, 8, 23]. Furthermore, it belongs to the most malaria-affected natural region in the country where the prevalence of malaria among children under 5 years of age is 61% [12]. Guéckédou district consists of 10 sub-districts and an urban commune.

The study sites specifically included the sub-district of Guèndembou, which had the highest EVD case burden in Guéckédou (60 reported cases), and the sub-district of Bolodou, which was less affected by the EVD outbreak (1 reported case) [2]. At the time of the study (post-EVD period) Guèndembou had one health centre, one private clinic, and six health posts for target under-five population of 6281 people. In Bolodou, there were one health centre and four health posts, for a target under-five population of 2729 people. The number of health facilities available and functional in the post-EVD period was similar to the number in the pre-EVD context [source: Guéckédou Health District Office, 2017].

### Operational definition of health-seeking behaviour and conceptual framework

We defined health-seeking behaviour as a “sequence of remedial actions that individuals undertake to rectify perceived ill-health” [24]. In this study, we focused on caregivers’ decisions regarding the type of healthcare provider patients sought help from, reasons for choice of healthcare professional, and reasons for not seeking help from healthcare professionals [24].

**Fig. 2:**
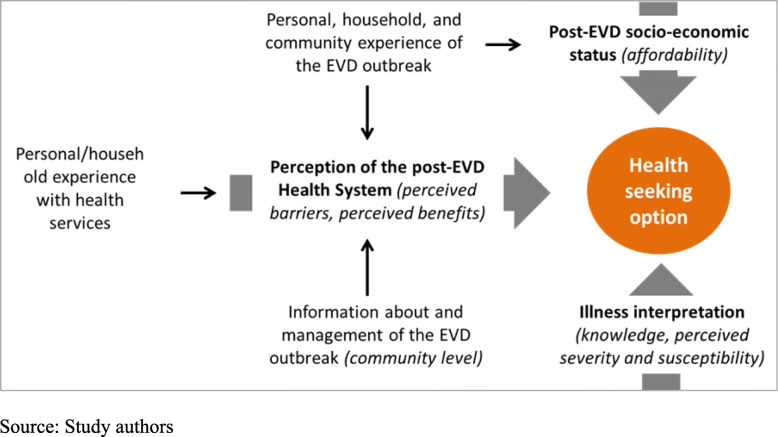
Conceptual framework of factors influencing post-EVD care seeking behaviors. Source: Study authors

Selection of our study variables was based on a conceptual framework produced by adapting Metta’s model and the Partners for Applied Social Sciences (PASS) model, as well as accounting for authors (BSC and AD)‘s community experience of the EVD outbreak (Fig. 2) [25, 26]. It draws on the pattern of post-EVD outbreak (January 2016–September 2017) health-seeking behaviours as interplay among three main aspects: the perception of the post-EVD health system, post-EVD socio-economic status, and illness interpretation. The perception of post-EVD health system conditions and post-EVD care-seeking behaviour is informed by i) messages and rumours at the community level about the EVD outbreak and its management by the health system; ii) personal, household, or community experience of the EVD outbreak; and iii) personal experience with health services. These factors could shape individuals’ perceived barriers or benefits (e.g., risk of EVD contamination at facility; availability of services; belief in care providers; their attitudes; quality and cost of care) and make them seek care through a given health service channel. Post-EVD socioeconomic status can be affected by the personal or household experience of the EVD outbreak (e.g., if the EVD death concerns a productive or supportive member of the household). Illness interpretation also guides individuals’ decision to resort to a given health service channel. It depends mainly on individuals’ knowledge of the illness, their perceived severity, and their perceived susceptibility to being at risk of the illness.

### Study participants and sampling

Caregivers of children under 5 years of age were surveyed. We considered as caregiver the child’s mother or the main person caring for him or her at home. Caregivers were selected through two-stage cluster sampling. In the first stage, the sub-district with the highest reported EVD case burden (Guèndembou; 60 cases) in Guéckédou and a sub-district with a low reported EVD case burden (Bolodou; one case) were selected. For the second stage, in each selected sub-district, all households with a child aged less than 5 years who had a fever episode (as reported by the caregiver) within the preceding 30 days were included in the study. The caregiver of one eligible child per household was selected. In households with more than one eligible child, the child who had the most recent episode was selected. All households were visited with the help of local guides, moving from the middle to the ends of each village, clockwise. The visiting process was proceeded from the main village to the surrounding villages of each sub-district, clockwise, until the desired sample size was reached. In total, 14 villages (seven in each sub-district) were visited.

The sample size was calculated using Cochran’s sample size formula, which is appropriate for cross-sectional studies [27]. It was based on the proportion of febrile children for whom caregivers sought health services in Guinea in 2012 (37%) [12], a confidence level of 95% and a margin error of 5%. A minimum of 358 caregivers were needed for the study. Half of the study participants were expected to come from each sub-district, to account for actual situation in each of the two sub-district.

### Data collection and variables

Data were collected from 24 September to 4 October 2017 by trained surveyors using Open Data Kit (ODK) with Android mobile phones. The survey questions were specifically developed for the purpose of this study.

The study variables included outcome and independent variables. The outcome variable was seeking care for the under-five febrile child (sought care? Yes = 1, No = 2). Additional variables were described in the sub-study population which sought care for the under-five febrile child. They included health-seeking place, reasons for selecting the health-seeking place); utilization of health services (blood test performed [yes/no], medicines given [yes/no]). Independent variables included EVD-related events and feelings (occurrence of EVD deaths in the household/family, whether EVD impoverished the household/family, fear of shaking hands with friends, fear of hugging friends, fear of sharing plates with friends, fear of hugging household/family members, fear of sharing plates with household/family members, fear of kissing household/family members, preference for washing hands with chlorine solution, keeping chlorine solution at home); interpretation of the child’s illness (the child could eat or breastfeed as usual [yes/no], the child could move as usual [yes/no], meaning of the illness to the caregiver, diagnosis of the illness); and perception of service quality at health facilities as compared with pre-EVD (medicine availability, antimalarial drug availability, availability of rapid test kits for malaria, waiting time, staff listening to patients, cases left without treatment, staff reliability, facility cleanliness, cost of care, quality of treatment). The sociodemographic characteristics of caregivers and their children were also assessed as covariates for the outcome variable. They included caregiver’s age, education level, marital status, number of household members, main source of household income, household characteristics, and age of the child, and gender of the child.

### Data analysis

Descriptive variables were presented as proportions or means with standard deviations (SD). Numerical values were assigned to household characteristics by adapting the method developed by the health and demographic survey [12] to measure household property scores. EVD-related feelings were also assigned numerical values to measure EVD outbreak fear among caregivers. Caregivers’ level of fear was as assessed using numerical scores, and the maximum fear score had a value of 10. Pearson’s chi-square (*X*^2^) and student t-tests were used to compare the variables between the two sub-districts.

A logistic regression using a backward stepwise model was conducted to predict care seeking behaviour. Adjusted odds ratios (AOR) were then derived with 95% confidence intervals (CIs). The level of significance was set at *p* < 0.05.

Data were analysed using SPSS software version 22.0 for Windows (SPSS Inc., Chicago, IL, United States).

## Results

### Sociodemographic characteristics of respondents

The number of households visited in Guèndembou and Bolodou were 219 and 241 respectively. We found eligible children in 401 of the eligible households (198 in Guèndembou and in 203 in Bolodou).

Overall, 398 caregivers were surveyed. Non response rate was less than 1% (*n* = 3). The sociodemographic characteristics of surveyed caregivers differed between the two sub-districts with the exception of the number of household members (Table 1). Caregivers living in Guèndembou had a higher mean age (33·9 years; SD: 13·9 years) than those in Bolodou (28·7 years; SD: 7·2 years; *p* < 0·001). They were predominantly married or in a union, with a significantly higher proportion of such people in Bolodou (92·1% (*n* = 186) compared to 84·7% (*n* = 166) in Guèndembou; *p* = 0·021). Caregivers in Guèndembou had higher household property mean score (17.6; SD: 5.1) than those living in Bolodou (16.0; SD: 4.7). In Guèndembou, the majority (58·7%; *n* = 115) of caregivers had attended at least primary school, whereas in Bolodou, most caregivers had no education at all (73·8%; *n* = 149; *p* < 0·001).

**Table 1:** Sociodemographic characteristics of febrile children’s caregivers in Guèndembou and Bolodou sub-districts, September–October 2017, Guinea

			(*N* = 398)
	Guèndembou (***N*** = 196)	Bolodou (***N*** = 202)	***p*** value^**a**^
Age of respondents			< 0·001
Mean (SD) years	33·9 (13.9)	28·7 (7.2)	
Sex of child			0·021
Male (%)	89 (45·4%)	115 (56·9%)	
Female (%)	107 (55·6%)	87 (43·1%)	
Age of child			0·002
Mean (95% CI) months	28·3 (+/−15·4)	33·3 (+/−16·3)	
Educational level			< 0·001
None (%)	81 (41·3%)	149 (73·8%)	
Incomplete primary school (%)	72 (36·7%)	43 (21·3%)	
Primary school or more (%)	43 (22·0%)	10 (4·9%)	
Marital status			0·021
Married/in union (%)	166 (84·7%)	186 (92·1%)	
Not married (%)	30 (15·3%)	16 (7·9%)	
Number of household members			0·072
Mean (95% CI) persons	7·7 (+/−2·8)	7·2 (+/−2·7)	
Household property score^b^			
Mean (95% CI) points	17·6 (+/−5·1)	16·0 (+/−4·7)	0·001
Above median (≥15.0) (%)	115 (58·7%)	74 (36·6%)	< 0·001
Source of income			< 0·.001
Farming (%)	167 (85·2%)	195 (96·5%)	
Street vendor (%)	17 (8·7%)	2 (1·0%)	
Other (%)	12 (6·1%)	5 (2·5%)	

^a^Chi-square test or student t-test

^b^Full score of the household property score was 62 points, and median of all the studied households was 15 points

### EVD- related events and feelings

More caregivers in Guèndembou (19·9%; *n* = 39) reported the death of family members or friends due to EVD compared to Bolodou (6·9%; *n* = 14; *P* < 0·001) (Table 2). The majority of participants in both districts felt that the EVD outbreak had impoverished their families, with a higher proportion in Bolodou (76·2% (*n* = 154) compared to 64·3% (*n* = 126) in Guèndembou; *p* = 0·009).

**Table 2:** EVD-related events and feelings among caregivers in Guèndembou and Bolodou sub-districts, September–October 2017, Guinea

			(*N* = 398)
	Guèndembou (***N*** = 196)	Bolodou (***N*** = 202)	***p*** value^**a**^
Reported EVD cases during the period of 2013–2015	60	1	n.a.
Family/friends’ death due to EVD			
Yes (%)	39 (19·9%)	14 (6·9%)	< 0·001
EVD outbreak impoverished family/household			
Yes (%)	126 (64·3%)	154 (76·2%)	0·009
Fear of shaking hands with friends			
Yes (%)	50 (25·5%)	22 (10·9%)	< 0·001
Fear of hugging friends			
Yes (%)	44 (22·5%)	19 (9·4%)	< 0·.001
Fear of sharing plates with friends			
Yes (%)	44 (22·5%)	15 (7·4%)	< 0·001
Fear of hugging family members			
Yes (%)	32 (16·8%)	2 (1·0%)	< 0·001
Fear of sharing plates with family/household members			
Yes (%)	33 (16·8%)	2 (1·0%)	< 0·001
Fear of kissing family/household members			
Yes (%)	35 (17·9%)	1 (0·5%)	< 0·001
Fear of sharing bed or bed linen with family/household members			
Yes (%)	34 (17·3%)	2 (1·0%)	< 0·001
Prefer to wash hands with chlorine solution			
Yes (%)	126 (64·3%)	178 (88·1%)	< 0·001
Keeping chlorine at home			
Yes (%)	109 (55·6%)	18 (8·9%)	< 0·001
Fear score^b^			
Mean points (95% CI)	3·0 (2·6–3·4)	2·.0 (1·8–2·1)	< 0·001
Above median (≥2.0)^c^ (%)	81 (41·3%)	31 (15·3%)	< 0·001

*n.a.* not applicable

^a^Chi-square test or student t-test

^b^Fear score is calculated by summing the number of ten fear-related questions to which participants agreed; hence, the score ranges from 0 to 10

^c^Frequency of individual fear scores is equal to or more than the median of whole sample

Additionally, more caregivers in Bolodou (88·1%; *n* = 178) said they preferred washing their hands with chlorine solution than in Guèndembou (64·3%; *n* = 126; *p* < 0·001); however, the number of those who claimed to keep chlorine solution at home were far more in Guèndembou (55·6%; *n* = 109) than in Bolodou (8·9%; *n* = 18; *p* < 0·001). In Guèndembou significantly more respondents reported being fearful of shaking hands with friends (25·5%; *n* = 50) or hugging friends (22·5%; *n* = 44) as compared to 10·9% (*n* = 22) and 9·4% (*n* = 19) in Bolodou (*p* < 0·001), respectively. Significantly more caregivers in Guèndembou said that they were fearful of sharing a bed or bed linen (17·3%; *n* = 34; *p* < 0·001) or plates (16·8%; *n* = 33; *p* < 0·001) with family members, or of hugging them (16·8%; *n* = 32; *p* < 0·001), while the three proportions were all 1% (*n* = 2; *p* < 0·001) in Bolodou. The mean fear score of caregivers was significantly higher in Guèndembou (3·0; SD: 3·0) than in Bolodou (2·0; SD: 1·1) (*p* < 0·001).

### Health-seeking options and reasons

More caregivers sought health care for children with febrile illnesses in the EVD-affected sub-district, i.e., Guèndembou (80·6%; *n* = 158) than in Bolodou (68·8%; *n* = 139; *p* = 0·007) (Table 3). In Guèndembou, 105 of 158 (66·5%) respondents who sought care did so at public health facilities, while in Bolodou 128 of 139 (92·1%) did so (*p* < 0·001).

**Table 3:** Illness interpretation and health services use in Guèndembou and Bolodou sub-districts, September–October 2017, Guinea

			(*N* = 398)
	**Guèndembou (*N*** **= 196)**	**Bolodou (*N*** **= 202)**	***p*** **value**^**a**^
The child could not eat or breastfeed as usual			0·374
Yes (%)	98 (50·0%)	110 (54·5%)	
The child could not move as usual			< 0·001
Yes (%)	50 (25·5%)	124 (61·4%)	
Caregiver’s interpretation of the illness			0.·002
Malaria (%)	150 (76·5%)	149 (73·8%)	
Cold (%)	15 (7·7%)	37 (18·3%)	
Teething (%)	7 (3·6%)	2 (1·0%)	
Other^b^ (%)	24 (12·2%)	14 (6·9%)	
Sought care			0·007
Yes (%)	158 (80·6%)	139 (68·8%)	
**Only respondents who sought care**	**Guèndembou (*N*** **= 158)**	**Bolodou (*N*** **= 139)**	
Places of care			< 0·001
Public health facility^c^ (%)	105 (66·5%)	128 (92·1%)	
Private clinic (%)	42 (26·5%)	2 (1·4%)	
Others^d^ (%)	11 (7·0%)	9 (6·5%)	
Reasons for care place selection			
Affordable cost (%)	90 (57·0%)	13 (9·4%)	< 0·001
Affordable transportation (%)	85 (53·8%)	12 (8·6%)	< 0·001
Available transportation (%)	94 (59·5%)	11 (7·9%)	< 0·001
Accessible distance (%)	64 (40·5%)	17 (12·2%)	< 0·001
Convenient open hours (%)	105 (66·5%)	52 37·4%)	< 0·001
Available staff (%)	112 (70·9%)	66 (47·5%)	< 0·001
Available medicines (%)	146 (92·4%)	135 (97·1%)	0·072
Short waiting time (%)	110 (79·6%)	41 (29·5%)	< 0·001
Good staff attitude (%)	134 (84·8%)	62 (44·6%)	< 0·001
Low risk of EVD transmission (%)	80 (50·6%)	89 (64·0%)	0·020
Low risk of misdiagnosis (%)	81 (51·3%)	88 (63·3%)	0·036
Blood test			0·031
Yes (%)	131 (82·9%)	127 (91·4%)	
Diagnosed as malaria			< 0·001
Yes (%)	144 (91·1%)	103 (74·1%)	
Medicines given			0·183
Yes (%)	156 (98·7%)	139 (100·0%)	

^a^Chi-square test

^b^Fever, headache, freshness, infantile shock, mystical disease, don’t know

^c^Health centre/post or district hospital

^d^Drug shop or open vendor, traditional healer

The primary reason for selecting care facilities was the availability of medicines in both sub-districts (92·4% (*n* = 146) in Guèndembou and 97·1% (*n* = 135) in Bolodou). The other main reasons in Guèndembou were good staff attitude (84·.8%; *n* = 134), availability of staff (70·9%; *n* = 112), and a short waiting time (79·6%; *n* = 110), while the main reasons in Bolodou were low risk of EVD transmission (64·0%; *n* = 89) and low risk of EVD misdiagnosis (63·3%; *n* = 88).

### Perception of health service quality compared with the pre-EVD period

The majority of caregivers in Guèndembou (82·1%; *n* = 161) agreed that more medicines were available at the facilities than during the pre-EVD period, whereas only 29·7% (*n* = 60; *p* < 0·001) agreed with this in Bolodou (Table 4). Opinions regarding health staff availability differed across the two sub-districts; whereas 85·7% (*n* = 168) of caregivers agreed that more health staff was available at facilities in Guèndembou, only 35·6% (*n* = 72; *p* < 0·001)) agreed with this in Bolodou. In Guèndembou, 70 of 196 (35·7%) respondents agreed that the cost of health care was less expensive than during the pre-EVD period, whereas only 14 of 202 (6·9%) in Bolodou agreed with this (*p* < 0·001).

**Table 4:** Perceived health service quality compared with pre-EVD outbreak in Guèndembou and Bolodou sub-districts, September–October 2017, Guinea

			(*N* = 398)
	Guèndembou (***N*** = 196)	Bolodou (***N*** = 202)	***p*** value^**g**^
More medicines are available			< 0·001
Agree (%)	161 (82·1%)	60 (29·7%)	
More antimalarial medicines are available			0·011
Agree (%)	171 (87·2%)	191 (94·6%)	
More rapid test kits for malaria diagnosis are available			0·002
Agree (%)	184 (93·9%)	201 (99·5%)	
More health staff is available			< 0·001
Agree (%)	168 (85·7%)	72 (35·6%)	
Shorter waiting time			0·008
Agree (%)	135 (68·9%)	113 (55·9%)	
Health staff spend more time listening to patients			< 0·001
Agree (%)	164 (83·7%)	98 (48·5%)	
Fewer cases are left without treatment			< 0·001
Agree (%)	189 (96·4%)	127 (62·9%)	
Health staff is more reliable			< 0·001
Agree (%)	157 (80·1%)	114 (56·4%)	
Health facilities are cleaner			0·002
Agree (%)	194 (99·0%)	186 (92·1%)	
Cost of care is less expensive			< 0·001
Agree (%)	70 (35·7%)	14 (6·9%)	
Better treatments are given			< 0·001
Agree (%)	177 (90·3%)	121 (59·9%)	

^g^Chi-square test

### Factors associated with seeking care

A bivariate analysis was conducted to examine factors that were associated with seeking health care for children under-5 years of age (Table 5). Factors that were significantly associated with seeking care included residing in Guèndembou, death of a family member or friend due to due to EVD, perceived greater quantity of medicines at health facilities in the post-EVD period, perceived better treatment at health facilities post-EVD, and perceived less cost of care at health facilities post-EVD. Caregivers with a fear score above the median were 1·68 times more likely to seek care than those whose fear score was equal to or below the median; however, this difference was not statistically significant.

**Table 5:** Factors associated with seeking care for under-five fever in Guèndembou and Bolodou sub-districts, September–October 2017, Guinea

	Sought care for children		
	***n*** = 297	Odds ratio (95%CI)	***P***-value
Residing sub-district			
Guèndembou (*n* = 196)	158 (80·6%)	1·89 (1·19–2·99)	*p* = 0·007
Bolodou (*n* = 202)	139 (68·8%)	1	
Household property score			
Above median (*n* = 189)	148 (78·3%)	1·45 (0·92–2·30)	n.s*.*
Below median (*n* = 209)	149 (71·3%)	1	
Family/friends’ death due to EVD			
Yes (*n* = 53)	46 (86·8%)	2·46 (1·07–5·64)	*p* = 0·029
No (*n* = 345)	251 (72·8%)	1	
The child could not eat/breastfeed as usual			
Yes (*n* = 190)	137 (72·1%)	1	
No (*n* = 208)	160 (76·9%)	1·29 (0·82–2·03)	n.s.
The child could not move as usual			
Yes (*n* = 190)	168 (88·4%)	1	
No (*n* = 208)	129 (62·0%)	0·96 (0·61–1·51)	n.s.
More medicines are available at health facilities			
Agree (*n* = 221)	175 (79·2%)	1·72 (1·09–2·07)	*p* = 0·019
Disagree (*n* = 177)	122 (68·9%)	1	
Better treatments are given at health facilities			
Agree (*n* = 298)	232 (77·9%)	1·89 (1·16–3·10)	*p* = 0·011
Disagree (*n* = 100)	65 (65·0%)	1	
Health staff listens more to patients at health facilities			
Agree (*n* = 262)	200 (76·3%)	1·30 (0·81–2·07)	n.s.
Disagree (*n* = 136)	97 (71·3%)	1	
Cost of care is less expensive at health facilities			
Agree (*n* = 84)	71 (84·5%)	2·13 (1·12–4·04)	*p* = 0·019
Disagree (*n* = 314)	226 (72·0%)	1	
Fear score			
Above median (*n* = 112)	91 (81·3%)	1·68 (0·98–2·89)	n.s.
Below median (*n* = 286)	206 (72·0%)	1	

*n.s.* not statistically significant

^a^Above the median score

In the multiple-logistic regression analysis (Table 6), only residing in Guèndembou (AOR = 1·74; 95%CI: 1·09–2·79) was positively associated with seeking care. Caregivers who reported family members’ or friends’ death due to EVD were also more likely to seek care (AOR = 2·12; 95%CI: 0·91–4·91), however, with no statistical significance.

**Table 6:** Logistic regression^a^ to predict seeking care for under-five fever in Guèndembou and Bolodou sub-districts, September–October 2017, Guinea

						*N* = 398
Variable	Coefficient (β)	Standard error	Wald χ^**2**^	***P*** value	Odds ratio	(95%CI)
Intercept	0·747	0·154	–	–	–	–
Residing in Guèndembou	0·555	0·240	5·375	0·020	1·743	1·090–2·787
Family/friends’ death due to EVD	0·750	0·430	3·044	0·081	2·117	0·912–4·914

Variables input: Residing in Guèndembou, above median household property score, family/friends’ death due to EVD, current perceptions of quality of the public health facility: more medicines, health staff listening more to patients, and above median fear score

^a^ Backward stepwise model

## Discussion

This study reveals that a number of community members in the rural district of Guéckédou still live with fear related to EVD nearly 2 years after the outbreak. As expected, more family members’ or friends’ deaths due to EVD were reported in the EVD-most affected sub-district than the less affected sub-district. However, this did not prevent utilizations of health services if a child under 5 was febrile.

Indeed, we were expecting to find a negative impact of the EVD memories on post-outbreak health-seeking behaviour for febrile illnesses. Previous studies on post-EVD health service utilization in Guinea reported recovery gaps demonstrating that health service utilization was lower in 2016 compared to the pre-EVD period [9, 16–18]. The present findings reflect two main assumptions regarding post-EVD health service utilization. First, these findings might indicate gradual improvement in health service utilization after the EVD outbreak. Dunbar et al. reported that the malaria program in Liberia could require 26 months after the acute phase of the EVD outbreak to recover to pre-EVD levels [28]. Improvement in utilization of health services could be explained by the perceived improvement of health service quality by communities, as shown by this study, in the post-EVD compared to the pre-EVD periods. Indeed, with the post-outbreak momentum to improve a health system that had been shaken by a recent history of community mistrust due to the outbreak, it is likely that priority would be given to the rebuilding of the community’s trust in the health system. The second assumption is that recovery gap in post-EVD health service utilization reported in Guinea [9, 16–18] might be related to other factors, such as health system factors or other community factors. Qualitative research could better contribute to explain the difference between pre- and post-EVD outbreak health seeking behaviour. However, the absence of pre-EVD indicators on health service utilization in the two sub-districts constitutes a major limitation to drawing conclusion on the influence of EVD memories on post-EVD health service utilization. We could not access reliable data on indicators such as facility attendance or quality of services in our study settings; many variables of interest including symptoms, body temperature measurement, malaria testing, treatment, were missing for most under-five children. However, assuming that health service users’ opinions on quality of services have an influence on their health seeking behaviours, we accounted for users’ opinions on service quality in the post-EVD compared to the pre-EVD period, to assess association between EVD memories and health service utilization.

Caregivers living in the EVD-affected sub-district of Guèndembou were more likely to seek care for their febrile child than caregivers living in the less affected sub-district of Bolodou. The possible factor favouring health service utilization in Guèndembou could be the presence of a private clinic that offers the community the opportunity to receive care on credit, unlike in Bolodou, where no private clinic existed. The private clinic in Guèndembou was owned by a retired nurse who was told by caregivers to be renown in the locality as ‘good and kind doctor’. He had been providing private health services in the sub-district for nearly 10 years. We found that 33.5% of caregivers in Guèndembou sought care in this private clinic. This emphasizes disparities in access to health services across sub-districts in the country, and how these shape health seeking behaviours. Health disparities across local settings have been reported to often be hidden by the improvements shown by national/global indicators [29]. This result may also suggest the possibility of a greater commitment of community actors to increasing community awareness to utilise health services, and of health workers to improving health service quality in the EVD-affected sub-district. Improvement of health services following the outbreak was perceived by more caregivers in Guèndembou (most affected by EVD) than Bolodou (less affected). In addition, we explored whether any post-EVD initiative targeting the EVD-affected sub-district occurred in the study setting before our survey, to which improved utilization of health services could be attributed. However, no particular initiative occurred at that time.

The study findings also showed positive and negative influences of the EVD outbreak on community members’ post-EVD attitudes. As positive influence, people were more likely to utilize chlorine for hand hygiene. In fact, more than half of the caregivers in Guèndembou keep chlorine at home, implying that these individuals are aware of its importance. Furthermore, the majority of caregivers in both sub-districts preferred to wash their hands with chlorine. Such attitude could result from greater chlorine stocks left over in Guèndembou from community infection prevention interventions during the outbreak. It could also reflect a positive perception of rural communities toward chlorine, thereby offering an opportunity to further encourage its use for infection prevention at community level. However, Somparé questioned the community’s will to continue systematic hand washing with chlorine in the long run given that this practice might trigger negative memories of the EVD outbreak. Some community members might even reject any symbolic behaviour or practices related to EVD [30]. This suggests the need for appropriate strategies involving social and health scientists to sustain infection prevention practices implemented in communities during the EVD crisis. Effective infection prevention measures such as use of soap and water would therefore be appropriate in this context since such measure could be acceptable and feasible locally.

As a negative influence, we found that residents of Guinean rural communities live with a persistent fear of EVD contamination that affects their social relationships. For instance, despite this being one of the community’s key values, a number of caregivers refrain from shaking hands with or hugging friends or family members. In a context where cultural values prevail as is the case in Guinea [31], such attitudes reflect a sociocultural impact of the EVD outbreak and are more likely to weaken social ties in rural Guinea. In addition, this could reflect a psychosocial effect of EVD on individuals since people’s negative memories of EVD-related deaths and stigma could drive such attitudes. This suggests the need for refined health education strategies at the community level in a manner that contributes to preserving the community’s key values. It also calls for further research on the psychosocial effects of EVD in Guinean communities in order to restore individuals’ sense of psychosocial well-being. Shanahan reported that many bereaved people and orphans from the EVD outbreak were exposed to complex grief and stress after the outbreak in Sierra Leone and urged health workers to address their psychosocial needs [32].

Surprisingly, more participants in the EVD less affected sub-district of Bolodou felt that the EVD outbreak had impoverished their families, than participants in the EVD most affected sub-district of Guèndembou. A survey across Guinea in 2015 reported a greater than 30% decline in income for rural households, however in areas severely affected by the EVD outbreak [33]. Qualitative study with some of this study participants showed that the EVD economic impact on households in the study settings was mainly due to a slowdown in their economic activities. Indeed, trade activities across villages were restricted during the outbreak, resulting in waste of agricultural products, which constituted the main source of community’s economy. The EVD less affected sub-district of Bolodou might have been more affected economically because its community lives more from agriculture than in Guèndembou. Our findings showed that farming is the main source of income for 96.5% of respondents in Bolodou, compared to 85.2% in Guèndembou. What’s more qualitative findings showed that in the district of Guéckedou, bigger sub-districts such as Guèndembou and Guéckédou town constituted the main trading points for villages, while these points where among the EVD most affected areas. Farmers from Bolodou might have therefore refrained from travelling to EVD high risk areas to prevent EVD contamination, resulting in more waste of their agricultural products.

Some limitations to this study should be noted. First, the study being cross-sectional and conducted in the post-EVD, we could not access reliable pre-EVD data on health seeking behaviours of our study population to better understand EVD-attributed influence on their health seeking behaviours. We expected to analyse - as proxy – routine data on health facility attendance in the study sub-districts; however these data had considerable missing information on our variables of interest. Second, the comparison groups—the sub-districts as well as caregivers across the sub-districts—were not directly comparable; this created the risk of potential bias in estimating the effect of the EVD outbreak on health-seeking behaviours. Indeed, Guendembou is bigger in terms of surface area and population size than Bolodou, with more health facilities including a private clinic; this could favour utilization of health services. Analysis of the study population socio-demographic characteristics also showed statistical difference in education level and household property score across the two sub-districts, which we thought to also have an influence on our outcome of interest, i.e., health service utilization. In addition, any EVD-independent change in terms of quality and or access to health services in the post-EVD compared to the pre-EVD across the two sub-district could influence our outcome of interest. However, this study has the privilege to report insights from health service users on the influence of the EVD outbreak on their health seeking behaviours, but also account for identified covariates at the analysis level. Third, the study was limited to two sub-districts within a single district and therefore could not address the situation in the other EVD-affected districts in the country. However, our data allowed for a comparison between an EVD-affected sub-district and the least affected sub-district. The study should therefore be relevant for districts with similar EVD epidemiological contexts. In addition, one strength of this study is that it is lines with the STROBE items that should be included in reports of cross-sectional studies (Additional file 1).

## Conclusions

This study found that community members in the rural district of Guéckédou still live with fear related to EVD nearly 2 years after the outbreak. However, it demonstrated that EVD memories had no influence on utilization of health services for under-five febrile illnesses, nearly 2 years after the outbreak. In addition, findings showed use of chlorine for hand hygiene as a community’s preferred infection prevention measure in the EVD-affected sub-district. This study also calls for more efforts in the health domain to preserve communities’ key values and address the psychosocial effect of EVD in rural Guinea.

## Supplementary information

**Additional file 1.** STROBE Guidelines checklist. STROBE Statement—Checklist of items that should be included in reports of cross-sectional studies. This checklist is a table giving pages and lines numbers of this paper where items that should be included in reports of cross-sectional studies, are mentioned.

## Data Availability

The questionnaire and datasets used and/or analysed during the current study are available and can be accessed from the corresponding author on reasonable request.

## Abbreviations

AOR: Ajusted odd ratio
CHWs: Community health workers
CI: Confidence interval
EVD: Ebola virus disease
SD: Standard deviation
