# Supplementary material for: Do memories of the Ebola virus disease outbreak influence post-Ebola health seeking behaviour in Guéckédou district (epicentre) in Guinea? A cross-sectional study of children with febrile illness
Source: BMC Public Health. 2020 Aug 27;20:1298. doi: 10.1186/s12889-020-09359-0 (PMC7450797; doi:10.1186/s12889-020-09359-0)
Supplement: Supplementary file 1 — Additional file 1. STROBE Guidelines checklist. STROBE Statement—Checklist of items that should be included in reports of cross-sectional studies. This checklist is a table giving pages and lines numbers of this paper where items that should be included in reports of cross-sectional studies, are mentioned. [file 12889_2020_9359_MOESM1_ESM.docx]

**APPENDIX**

STROBE Statement—Checklist of items that should be included in reports of ***cross-sectional studies***

|  | Item No | Recommendation |
| --- | --- | --- |
| **Title and abstract** | 1 | (*a*) Indicate the study’s design with a commonly used term in the title or the abstract **(lines 1-3, page 1)** |
|  |  | (*b*) Provide in the abstract an informative and balanced summary of what was done and what was found **(lines 32-47, page 2)** |
| Introduction | | |
| Background/rationale | 2 | Explain the scientific background **(lines 58-88, pages 3-4)** and rationale **(lines 89-103, pages 4-5)** for the investigation being reported |
| Objectives | 3 | State specific objectives, including any prespecified hypotheses **(lines 103-105, page 37)** |
| Methods | | |
| Study design | 4 | Present key elements of study design early in the paper **(lines 109-111, page 5)** |
| Setting | 5 | Describe the setting, locations, and relevant dates, including periods of recruitment, exposure, follow-up, and data collection **(lines 113-133, pages 5-6)** |
| Participants | 6 | (*a*) Give the eligibility criteria, and the sources and methods of selection of participants (**lines 157-174, pages 7-8)** |
| Variables | 7 | Clearly define all outcomes, exposures, predictors, potential confounders, and effect modifiers. Give diagnostic criteria, if applicable (**lines 178-196, page 8**) |
| Data sources/ measurement | 8* | For each variable of interest, give sources of data and details of methods of assessment (measurement). Describe comparability of assessment methods if there is more than one group **(line 157, page 7; lines 178-196, page 8; lines 198-207)** |
| Bias | 9 | Describe any efforts to address potential sources of bias **(lines 372-385, page 16)** |
| Study size | 10 | Explain how the study size was arrived at **(lines 169-174, pages 7-8)** |
| Quantitative variables | 11 | Explain how quantitative variables were handled in the analyses. If applicable, describe which groupings were chosen and why **(lines 198-207, page 9)** |
| Statistical methods | 12 | (*a*) Describe all statistical methods, including those used to control for confounding **(lines 198-207, page 9)** |
|  |  | (*b*) Describe any methods used to examine subgroups and interactions **(Not applicable)** |
|  |  | (*c*) Explain how missing data were addressed **(Not applicable)** |
|  |  | (*d*) If applicable, describe analytical methods taking account of sampling strategy **(lines 198-207, page 9)** |
|  |  | (*e*) Describe any sensitivity analyses **(Not applicable)** |
| Results | | |
| Participants | 13* | (a) Report numbers of individuals at each stage of study—eg numbers potentially eligible, examined for eligibility, confirmed eligible, included in the study, completing follow-up, and analysed **(Not applicable)** |
|  |  | (b) Give reasons for non-participation at each stage **(Not applicable)** |
|  |  | (c) Consider use of a flow diagram **(Not applicable)** |
| Descriptive data | 14* | (a) Give characteristics of study participants (eg demographic, clinical, social) and information on exposures and potential confounders (**lines 213-225, pages 9-10**) |
|  |  | (b) Indicate number of participants with missing data for each variable of interest (**line 216, page 9**) |
| Outcome data | 15* | Report numbers of outcome events or summary measures **(lines 227-260, pages 10-11)** |
| Main results | 16 | (*a*) Give unadjusted estimates and, if applicable, confounder-adjusted estimates and their precision (eg, 95% confidence interval). Make clear which confounders were adjusted for and why they were included (**lines 262-273, pages 11-12**) |
|  |  | (*b*) Report category boundaries when continuous variables were categorized **(Not applicable)** |
|  |  | (*c*) If relevant, consider translating estimates of relative risk into absolute risk for a meaningful time period **(Not applicable)** |
| Other analyses | 17 | Report other analyses done—eg analyses of subgroups and interactions, and sensitivity analyses **(Not applicable)** |
| Discussion | | |
| Key results | 18 | Summarise key results with reference to study objectives **(lines 276-280, page 12)** |
| Limitations | 19 | Discuss limitations of the study, taking into account sources of potential bias or imprecision. Discuss both direction and magnitude of any potential bias **(lines 369-389, page 16)** |
| Interpretation | 20 | Give a cautious overall interpretation of results considering objectives, limitations, multiplicity of analyses, results from similar studies, and other relevant evidence (**lines 281-368, pages 12-15**) |
| Generalisability | 21 | Discuss the generalisability (external validity) of the study results (**lines 385-389, page 16**) |
| Other information | | |
| Funding | 22 | Give the source of funding and the role of the funders for the present study and, if applicable, for the original study on which the present article is based (**lines 416-418, page 17**) |

*Give information separately for exposed and unexposed groups.

**Note:** An Explanation and Elaboration article discusses each checklist item and gives methodological background and published examples of transparent reporting. The STROBE checklist is best used in conjunction with this article (freely available on the Web sites of PLoS Medicine at http://www.plosmedicine.org/, Annals of Internal Medicine at http://www.annals.org/, and Epidemiology at http://www.epidem.com/). Information on the STROBE Initiative is available at www.strobe-statement.org.
